# Multi-Protection of DL0410 in Ameliorating Cognitive Defects in D-Galactose Induced Aging Mice

**DOI:** 10.3389/fnagi.2017.00409

**Published:** 2017-12-08

**Authors:** Wenwen Lian, Hao Jia, Lvjie Xu, Wei Zhou, De Kang, Ailin Liu, Guanhua Du

**Affiliations:** State Key Laboratory of Bioactive Substance and Function of Natural Medicines, Institute of Materia Medica, Chinese Academy of Medical Sciences and Peking Union Medical College, Beijing, China

**Keywords:** DL0410, Alzheimer’s disease, mitochondrion, oxidative stress, neuroinflammation, apoptosis, synaptic protection

## Abstract

D-galactose has been reported to accelerate senescence in rodents, accompanied by a decline in learning and memory. We used a model of D-galactose-induced amnesia for the efficacy evaluation and pharmacologic studies of active compounds against Alzheimer’s disease (AD). DL0410 is a potent inhibitor against acetylcholinesterase (AChE) and, in the present study, the effect of DL0410 was evaluated in this model. We found that DL0410 could significantly improve the learning and memory of D-galactose induced aging mice in a series of behavioral tests: novel-object recognition test, nest-building test, Morris water maze test and step-through test. Pharmacologic studies were conducted from several aspects: the cholinergic system, mitochondrial respiration, oxidative stress, neuroinflammation, apoptosis and synaptic loss. The acetylcholine level and AChE activity were not altered by D-galactose but were slightly affected by DL0410 in the brain. DL0410 could significantly improve decreased mitochondrial respiration in the NADH chain and FADH_2_ chain, and protect mitochondrial ultrastructure. DL0410 reduced the accumulation of advanced glycation end products (AGEs) and malondialdehyde (MDA) and increase the total antioxidant capability of the brain via an increase in activity of catalase, glutathione peroxidase (GPx) and superoxide dismutase (SOD). RAGE expression was inhibited by DL0410, followed by the decreased activation of astrocytes and microglia. Subsequent phosphorylation of NF-κB was also reversed by DL0410, with lower expression of cyclooxygenase-2 (COX2) and iNOS. With respect to apoptosis, the activation of caspase 3 and cleavage of PARP were downregulated significantly by DL0410, after the inhibition of phosphorylation of JNK induced by inflammation and oxidative stress. Synaptic protection by DL0410 was also demonstrated. These data suggest that mitochondrial protection has a primary role in the ameliorating effect of DL0410 on the impaired learning and memory, oxidative stress, inflammation, apoptosis and synaptic loss induced by D-galactose. DL0410 is a promising candidate for the treatment of aging-related AD, and this study lays an important foundation for its further research and development.

## Introduction

The 2015 report from the United Nations stated that the number of people aged >60 years will double to reach about 2.1 billion by 2050. Developing countries will account for a large proportion of this growth (Wyss-Coray, 2016). Every tissue and organ in an organism is affected by aging, but the brain is more susceptible to gradual loss of structure and function (Mrak et al., 1997). Therefore, neurodegenerative diseases such as Alzheimer’s disease (AD) can be a serious challenge for elderly people. AD is characterized by a loss of cognitive function. AD is the most common neurodegenerative disorder and affects about 13% of people aged >65 years and 50% of those aged >85 years (Clark and Kodadek, 2016). Familial Alzheimer’s disease (FAD) with early onset, is caused by gene mutations, and can be inherited. Sporadic Alzheimer’s disease (SAD) with late onset, is associated mainly with aging, and results from complex interactions between the environment, lifestyle and genes (Kim et al., 2014). The mitochondrial free radical theory of aging proposed by Harman (1956) has been accepted widely. Also, declining mitochondrial function accompanied with AD has been documented (Swerdlow and Khan, 2004; Kapogiannis and Mattson, 2011; López-Otín et al., 2013). In accordance with the mitochondrial free radical theory of aging, the subsequent abnormality in oxidative metabolism would produce excess reactive oxygen species (ROS), which damages proteins, lipids, and nucleic acids, and should be a vital pathophysiology in the progression of AD (Blass and Gibson, 1991; Cecchi et al., 2002; Sun et al., 2016; Tönnies and Trushina, 2017).

D-galactose administration has been shown to induce impairments in memory and cognition in mice accompanied with aging (Haider et al., 2015; Budni et al., 2016). Therefore, the model of D-galactose-induced amnesia has been used widely for pharmaceutical studies of anti-AD agents (Banji et al., 2013; Ali et al., 2015; Pourmemar et al., 2017). D-galactose is a reducing sugar which can be converted to galactitol after long-term administration at a high dose (Bosch, 2006). Galactitol also causes the production of a large amount of superoxide anions and oxygen radicals, which could result in direct damage to the structure and function of cells. Damage to mitochondria would hamper the normal electron-transfer chain and adenosine triphosphate (ATP) production in oxidative phosphorylation, which in turn promotes ROS production, leading to a vicious cycle (Long et al., 2007; Fukui and Moraes, 2008; Chang et al., 2014). In addition, D-galactose could form advanced glycation end products (AGEs) with proteins or peptides through non-enzymatic glycation. AGEs and ROS would induce obvious inflammation in the brain via NF-κB signaling pathway (Lu et al., 2010; Srikanth et al., 2011). Apoptosis and loss of neurons in the hippocampus and cortex has also been demonstrated, which could explain the degeneration of brain tissue induced by D-galactose (Ullah et al., 2015).

**Figure 1:**
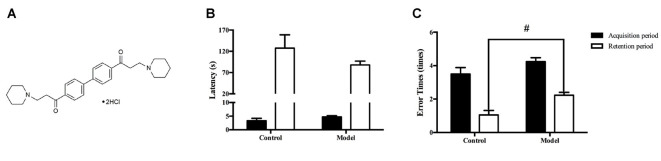
The chemical structure of DL0410 and administration of D-galactose for 6 weeks could lead to cognitive impairments. Data are the mean ± SEM (*n* = 20–120). **(A)** The chemical structure of DL0410. Administration of D-galactose for 6 weeks could lead to a shorter latency **(B)** and more error times (*t*_(140)_ = 2.159, *p* = 0.0326) **(C)** in the step-down test, ^#^*p* < 0.05 vs. control.

DL0410 (Figure 1A) is a potent acetylcholinesterase (AChE) inhibitor discovered in our previous virtual and high-throughput screening (Fang et al., 2015). In addition to its inhibition of AChE activity *in vitro*, the ameliorating effect of DL0410 on scopolamine-induced amnesia (a specific model mimicking changes in the cholinergic system) via the inhibition of AChE has been verified (Lian et al., 2017). In FAD-related mice models (APP/PS1 mice and an amyloid-beta (Aβ_1–42_)-induced amnesia model in mice), DL0410 showed a strong therapeutic effect against defects in memory and cognition, even better than that elicited by rivastigmine (positive control) at an identical dose (2 mg/kg). DL0410 not only inhibited AChE activity, it also inhibited Aβ_1–42_ production and promoted elimination of Aβ_1–42_, suggesting a key role in FAD treatment (Yang et al., 2015; Zhou et al., 2016). SAD comprise a majority of total AD, and SAD arises mainly from aging. However, the ameliorating effect of DL0410 on aging-related dementia has not been evaluated and novel mechanism deserves more efforts.

In the present study, a D-galactose-induced aging model, was set up as a representative model of SAD, and the improving effect of DL0410 was investigated. In addition to its effect on the cholinergic system, we also explored the mechanism of action from the viewpoint of D-galactose-induced mitochondrial damage and its potential pathway. In this way, our study could provide important information for the development of DL0410 as a candidate agent for aging-related AD.

## Materials and Methods

### Drugs and Reagents

DL0410 (purity ≥98% according to HPLC) was obtained from the Institute of Materia Medica, Chinese Academy of Medical Sciences (Beijing, China). Donepezil was purchased from Shandong Jinan Dexinjia Biotechnology (Jinan, China). Memantine was from Shanghai Jingchun Chemical Industry (Shanghai, China). D-galactose (V900922), adenosine 5′-diphosphate sodium salt (ADP, A2754) and succinic acid sodium (224731) were purchased from Sigma–Aldrich (St. Louis, MO, USA). Bovine serum albumin (BSA, 0332) and Tris-HCl (T22980) were obtained from Ameresco (Solon, OH, USA). L-glutamate, sucrose, ethylenediaminetetraacetic acid disodium salt dehydrate (EDTA·2Na), KH_2_PO_4_, Na_2_HPO_4_, KCl, NaCl and MgCl_2_·6H_2_O were purchased from Beijing Chemical Reagents Company (Beijing, China). L-malate (TM002601) was obtained from Sinopharm Chemical Reagents (Beijing, China). RIPA Buffer (#9806) and primary antibodies against cleaved caspase 3 (#9662), cyclo-oxygenase-2 (COX2, #12282) and inducible nitric oxide synthase (iNOS, #13120) were purchased from Cell Signaling Technology (Danvers, MA, USA). An Amplex Red Acetylcholine/Acetylcholinesterase Assay kit (A12217) was obtained from Invitrogen (Carlsbad, CA, USA). Primary antibodies against GAPDH (sc25778), receptor for advanced glycosylation end products (RAGE, sc365154), phosphorylated (p)-NF-κB P65 (sc33020), glial fibrillary acidic protein (GFAP, sc33673), Ionized calcium-binding adapter molecule 1 (Iba-1, sc98468), phosphorylated-c-Jun N-terminal kinase (p-JNK, sc6254) and poly(ADP-ribose) polymerase (PARP, sc7150) were purchased from Santa Cruz Biotechnology (Santa Cruz, CA, USA). A protease inhibitor cocktail (CW2200S), phosphatase inhibitor cocktail (CW2383S), bicinchoninic acid (BCA) Protein Assay kit (CW0014S), horseradish peroxidase (HRP)-conjugated secondary antibodies (anti-rabbit CW0234S, anti-mouse CW0221S), and ChemiGlow Western Blotting Detection Reagents (CW0049M) were purchased from Kangwei Biotechnology (Beijing, China). An advanced glycation end product (AGE)-competitive enzyme-linked immunosorbent assay (ELISA) kit (STA317) and thiobarbituric acid-reactive substances (TBARS) assay kit (for malondialdehyde (MDA) quantitation) (STA332) were obtained from Cell Biolabs (San Diego, CA, USA). Kits for total antioxidant capability (TAOC) assays (S0121), catalase assay (S0051), total glutathione peroxidase (GPx) assay (S0058) and total superoxide dismutase (SOD) assay with WST-8 (S0101) were purchased from Beyotime Biotechnology (Beijing, China).

### Animals and Treatments

All treatment and maintenance of animals was undertaken according to the *Guide for the care and use of laboratory animals* (National Institutes of Health, Bethesda, MD, USA) and approved by the Animal Care Committee of Peking Union Medical College and Chinese Academy of Medical Sciences (Beijing, China).

Male ICR mice (18–20 g, 8 weeks) were purchased from Vital River Laboratory Animal Technology (Beijing, China). Mice were raised in an environment at 23 ± 1°C and humidity of 50 ± 10%. Five mice per cage had free access to food and water under a controlled 12-h light–dark cycle (light on from 08:00 to 20:00). Mice were divided randomly into seven groups with 20 mice each group: control group, model group, DL0410-1 mg/kg group (DL-1 mg/kg group), DL0410-3 mg/kg group (DL-3 mg/kg group), DL0410-10 mg/kg group (DL-10 mg/kg group), Donepezil-3 mg/kg group (Don-3 mg/kg group), Memantine-3 mg/kg group (Mem-3 mg/kg group).

D-galactose was dissolved in sterile saline. DL0410, donepezil and memantine were dissolved in distilled water. They were given at 0.1 mL/10 g. In the first 6 weeks, mice in the control group were administered (s.c.) saline in the neck, and mice in the other six groups were administered D-galactose (180 mg/kg, s.c.) in the neck. At the end of the 6th week, the step-down test was used to confirm impairment of memory and recognition in mice treated with D-galactose (Figures 1B,C). From the 7th week, mice in the control group were given water (p.o.) and saline (s.c.) in the neck, mice in the model group were given water (p.o.) and D-galactose (s.c.) in the neck, and mice in the other five groups were given the corresponding drugs (p.o.) and D-galactose (s.c.) in the neck. Four weeks later, behavioral tests were conducted, and all tests were undertaken 30–40 min after drug administration. By the end of the behavioral tests, two mice in the control group, two mice in the model group, three mice in the Mem-3 mg/kg group, one mouse in the DL-1 mg/kg group, two mice in the DL-3 mg/kg group and two mice in the DL-10 mg/kg group had died.

### Behavioral Tests

#### Step-Down Test

The step-down test was conducted 6 weeks after D-galactose treatment. The step-down test was done in an apparatus (26 × 17 × 9 cm) with a metal floor connected to an electricity supply (36 V, 1.5 mA), and an insulated columnar platform fixed upon it. On the first day (acquisition phase), mice were put into the apparatus to accomodate for 1 min without electricity; then, mice were sited on the platform with electricity. Mice would step down to the floor and suffer an electric shock, and then they would climb onto the platform. Twenty-four hours later, retention tests were conducted using the same procedure. Each test lasted for 5 min, and two indices were recorded: latency (the time a mouse took to step down to the metal floor) and error time (total number of times a mouse stepped down to the metal floor; Yang et al., 2015).

#### Autonomous Activity Test

The autonomous activity test was conducted before all behavioral tests. The apparatus consists of a round box surrounded by six infrared detectors. A mouse was put into the box with an adaption time of 1 min, and then autonomous activity in 5 min was recorded. When the mouse moved from one place to another, detectors could detect the signal and record it as “one time”. The autonomous activity was the accumulation of detected signals in 5 min.

#### Novel Object Recognition Test

The novel-object recognition test was conducted in an open field box (50 × 50 × 40 cm). This test comprised three phases. In the first phase, mice were put in the box to accommodate for 5 min. Then, 24-h later, two red cubes (4 × 4 × 4 cm) were fixed in the paralleling in the box, 8 cm from the two nearest sides. Mice were placed in the box at the middle of the side far from objects, and the time that mice spent on exploring each object with their noses or paws (at a distance of ≤2 cm) was recorded. The third phase was conducted 24-h later, when one of the red cubes was replaced by a blue cone. The other process was the same as the second phase (Ennaceur and Delacour, 1988; Yang et al., 2015).

We used the Discrimination Index (DI) to evaluate the curiosity of mice towards new objects. The DI was defined as the percentage of time spent on a novel object (blue cone) to the total time spent on familiar (red cube) and novel objects.

#### Nest Building Test

The nest-building test was conducted in a cage (28 × 12 × 16 cm). Each mouse was assigned a separate cage for 24 h to accommodate. Before the night cycle, two stacks of tissue (4 × 4 cm, 6 pieces per stack) were placed in the cage. Nest-building results were awarded scores 12-h later using specific rules:

- 0, tissues were not touched at all;
- 1, tissues were scattered in the cage, but without being torn;
- 2, tissues were put together in a corner without being torn;
- 3, tissues were put together in a corner and a small part was torn, and a nest had not been formed;
- 4, most tissues were torn, and piled up in a corner, and a nest had obviously been made.

This test referred to the methods of Deacon, with some modifications (Gaskill et al., 2013; Otabi et al., 2016).

#### Morris Water Maze

The apparatus for a Morris water maze is composed of a black circular pool (diameter = 120 cm and height = 40 cm) and a platform (diameter = 8 cm, height = 20 cm). The pool was filled with water (23 ± 1°C). The platform was fixed in the center of the first quadrant and 1 cm below the water surface. The Morris water maze test lasted for 5 days. The first 4 days were navigation trials, and the last day was a probe trial.

In the navigation trial, mice were made to swim for 60 s twice in 1 day. The first-time mice were placed into the water in the middle of the third quadrant’s border facing the wall, and the second time in the middle of the second quadrant’s border. If mice found and stopped on the platform, the recording system would stop. Mice were allowed to stay on the platform for 15 s to consolidate memory. If a mouse could not find the platform within 60 s, it was guided to the platform and placed on the platform for 15 s.

In the probe trial, the platform was removed, and mice were allowed to swim for 60 s twice. The two sites were the same as those used for the navigation trial.

In this test, the swimming paths were recorded and analyzed. The performance on the fourth and fifth day was regarded as the final result of the navigation trial and probe trial, and used to assess the space memory (D’Hooge and De Deyn, 2001; Vorhees and Williams, 2006).

#### Step-Through Test

The apparatus for the step-through test consists of a transparent chamber (11.5 × 9.5 × 11 cm) and black chamber (23.5 × 9.5 × 11 cm) and a door connecting them. A metal floor in the black chamber is connected to an electricity supply (36 V, 1.5 mA). The transparent chamber is illuminated by a lamp.

In the acquisition phase, mice were placed in the transparent chamber facing a transparent well. Then, the power was turned on, and the door opened. When mice stepped into the black chamber, they would suffer an electric shock and return to the transparent chamber. This test lasted for 5 min. A retention test was conducted 24-h later, and mice went through the same process. During these two phases, latency (the time it took for mice to first step into the black chamber) and error time (the total time taken to step into the black chamber) were recorded (Zhou et al., 2016).

### Mitochondrial Respiration Assay

The separated cortex and hippocampus were homogenized immediately in cold separation medium (250 mM sucrose, 1 mM EDTA, 10 mM Tris, 0.1% BSA, pH 7.4) with a glass homogenizer on ice. The homogenate was centrifuged at 1000× *g* for 10 min at 4°C, and the supernatant was removed to a new Eppendorf tube. Then, the supernatant was centrifuged at 10,000× *g* for 10 min at 4°C. The supernatant was discarded, and the pellet was resuspended in 100 μL of cold separation medium. The protein concentration of an individual mitochondrion was determined using a BCA protein assay kit (Avetisyan et al., 2016).

A Clark oxygen electrode was used to evaluate mitochondrial respiration. First, 40 μL of an individual mitochondrion and 460 μL of respiratory medium were added into the reactor, and the baseline recorded. After the baseline had been stable for 1 min, 3 μL of L-glutamate (2 mol/L) and 3 μL of L-malate (1 mol/L) or 6 μL of succinate (1 mol/L) was added to induce a decrease in oxygen concentration. Then, 3 μL of ADP (50 mmol/L) was added to induce stage-3 respiration. When ADP was exhausted, stage-4 respiration appeared. The following indices were calculated: respiratory velocity of stage 3 (V3), V4, respiration control rate (RCR = V3/V4), and oxidative phosphorylation rate (OPR = V3 × ADP/O) (Zhang et al., 2017).

### Preparation of Brain Tissue

Thirty minutes after drug administration, mice were sacrificed. The cortex and hippocampus were collected and stored at −80°C.

In an assay to measure the level of ACh and activity of AChE, AGE, MDA and anti-oxidative enzymes, the cortex and hippocampus were homogenized together by Omni bead ruptor in 5 volumes of 0.01 M phosphate-buffered saline (PBS: 137 mM NaCl, 2.7 mM KCl, 10 mM Na_2_HPO_4_, 2 mM KH_2_PO_4_). Then, the homogenates were centrifuged at 12,000× *g* for 30 min at 4°C. The supernatant was collected and stored at −80°C for measurement.

Before western blotting, the cortex and hippocampus were homogenized, respectively, in five volumes of RIPA buffer supplemented with a protease inhibitor cocktail and phosphatase inhibitor cocktail. The supernatant was collected after centrifugation at 12,000× *g* for 30 min at 4°C. Loading buffer was added to the supernatant, which was then boiled for 10 min. The denatured protein was stored at −80°C. The protein concentration was determined by the BCA protein assay kit.

### Acetylcholinesterase (AChE) Activity and Acetylcholine (Ach) Assay

Measurement of AChE activity and Ach level was conducted in 96-well plates following the instructions of the Amplex Red Acetylcholine/Acetylcholinesterase Assay kit. For the assay of AChE activity, the reaction involved 100 μL of sample, 50 μM of Ach, 200 μM of Amplex Red Reagent, 0.1 U/mL of choline oxidase and 1 U/mL of HRP. The reaction was undertaken at room temperature for 20 min in the dark. Fluorescence intensity was measured at an excitation wavelength of 560 nm and emission wavelength of 580 nm.

The reaction system for ACh measurement was 100 μL of sample, 0.5 U/mL of AChE, 200 μM of Amplex Red Reagent, 0.1 U/mL of choline oxidase and 1 U/mL of HRP. The reaction system was incubated at room temperature for 30 min in the dark. Fluorescence intensity was measured at an excitation wavelength of 560 nm and emission wavelength of 580 nm (Park et al., 2016).

### Assay of Oxidation Related Biomarkers

#### ELISA Assay of AGE

The determination of AGE was conducted following the instructions of an OxiSelect™ Advanced Glycation End Product Competitive ELISA kit. Samples or AGE-BSA standards were added to wells coated with AGE conjugate, and incubated for 10 min at room temperature. Then, anti-AGE antibody was added, and the mixture incubated at room temperature for 1 h. After washing thrice, the secondary antibody-HRP conjugate was added and the mixture incubated for 1 h at room temperature. After washing thrice, the substrate solution was added. After 15 min of incubation at room temperature, stop solution was added to terminate the reaction. The absorbance was read at 450 nm. The AGE level was calculated according to the standard curve, and the AGE in the sample was expressed by the amount of AGE in 1 mg of protein.

#### MDA Assay

The MDA assay was conducted following a specific protocol. Briefly, 100 μL of the samples or MDA standards were added to microcentrifuge tubes, and 100 μL of sodium dodecyl sulfate lysis solution added and the mixture incubated at room temperature for 5 min. Then, 250 μL of thiobarbituric acid reagent was added to each tube, and the mixture incubated for 45 min at 95°C. The tubes were cooled to room temperature in an ice bath, and all tubes were centrifuged at 3000× *g* for 15 min. Then, 200 μL of the supernatant was transferred to a 96-well microplate, and the absorbance read at 532 nm. MDA in the sample was expressed by the amount of MDA in 1 mg of protein.

#### TAOC Assay

The TAOC assay was conducted following a specific protocol. Briefly, 20 μL of peroxidase solution was added to a 96-well microplate, and then 10 μL of the samples or Trolox standards added. Then, 170 μL of ABTS solution was added, and the reaction system incubated at room temperature for 6 min. The absorbance was read at 414 nm. The TAOC of the sample was represented by Trolox Equivalent Antioxidant Capacity (TEAC) in 1 mg of protein.

#### Assay of Catalase, GPx and SOD

The catalase assay was conducted following a specific protocol. Briefly, the samples, hydrogen peroxide (H_2_O_2_), and buffer were added to microcentrifuge tubes, and the system was incubated at room temperature for 4 min. Stop solution was added to terminate the reaction, and 10 μL added to another tube with detection buffer. Then, 10 μL of the system stated above was transferred to a 96-well microplate, and a colored solution added. The absorbance at 520 nm was read after incubation for 15 min at room temperature. Catalase activity in the sample was averaged by the concentration of protein.

The GPx assay was conducted following a specific protocol. Briefly, the sample, GPx detection solution, and detection buffer was added to a 96-well microplate. After H_2_O_2_ addition, the reaction was initiated. The absorbance at 340 nm was read at an interval of 30 s for 3 min continuously. GPx activity in the sample was averaged by the concentration of protein.

The SOD assay was conducted following a specific protocol. Briefly, samples, detection buffer, WST-8 solution and start solution were added to a 96-well microplate in order. The absorbance at 450 nm was read after incubation for 30 min at 37°C. SOD activity in the sample was averaged by the concentration of protein.

### Western Blotting

Ten percent SDS-PAGE was used to separate protein samples (about 80 μg protein), which were then transferred to PVDF membranes (Millipore, Billerica, MA, USA). PVDF membranes were then blocked by 5% BSA for 1 h at 37°C. PVDF membranes were cut off in accordance with the molecular weight, and incubated with different primary antibodies (rabbit anti-GAPDH, 1:1000 dilution; mouse anti-RAGE, 1:800; rabbit anti-p-NF-κB P65, 1:800; mouse anti-GFAP, 1:1000; rabbit anti-Iba-1, 1:800; mouse anti-p-JNK, 1:800; rabbit anti-PARP, 1:1000 from Santa Cruz Biotechnology; rabbit anti-COX2, 1:1000; rabbit anti-iNOS, 1:1000 from Cell Signaling Technology) at 4°C overnight. On the second day, PVDF membranes were incubated with HRP-conjugated secondary antibody (anti-mouse and anti-rabbit, 1:2000) for 2 h at 37°C. Protein bands were detected by an ECL western blotting kit using a ChemiDoc-It™ imaging system (UVP, Upland, CA, USA). GAPDH was selected as a control. The gray value was analyzed by Gel-pro 32 (Media Cybernetics, Rockville, MD, USA).

### Immunohistochemistry (IHC) Analysis

Three mice per group were selected at random and anesthetized by CO_2_ exposure. Perfusion was conducted with saline solution and 4% paraformaldehyde. Right brains were removed and fixed with 4% paraformaldehyde overnight. Brains were embedded with paraffin, cut into pieces of thickness 30 μm, and used for IHC staining.

Slides were dewaxed with xylene and a graded series of ethanol solutions. They were incubated with primary antibody (anti-GFAP and anti-Iba-1) overnight at 4°C. After rinsing with PBS, slides were incubated with secondary antibody for 1 h at room temperature. Then, they were visualized with diaminobenzidine using an Elite ABC kit (Vector, Burlingame, CA, USA). Hematoxylin was used for nuclear staining. For quantification of the mean integral optical density (IOD), three fields of the hippocampus or cortex were chosen for each slide. Each individual field was imaged at 400× magnification using a microscope and calculated using Image-Pro Plus 6.0 (Media Cybernetics, Rockville, MD, USA).

### Transmission Electron Microscopy (TEM)

After perfusion with saline solution and 4% paraformaldehyde, CA1 areas of the hippocampus in left brains were fixed with 4% paraformaldehyde for 2 h. Then, they were placed in 2.5% glutaraldehyde overnight at 4°C, followed by washing with 0.01 M PBS and preservation in 0.01 M PBS at 4°C. They were dehydrated with a graded series of alcohol solutions, embedded in Epon resin, and cut into pieces of thickness 50–60 nm. Slides were stained with uranyl acetate and lead citrate and observed under a transmission electron microscope (H-7650; Hitachi, Tokyo, Japan). For the ultrastructure of mitochondria, images were observed at 150,000× magnification. For the quantification of synapses, 10–15 fields were chosen randomly for each slide at 50,000× magnification.

### Statistics

Data are expressed as the mean ± SEM. Statistical analysis were conducted in GraphPad Prism software (San Diego, CA, USA). Data for the navigation trial of the Morris water maze was analyzed with two-way ANOVA with repeated measures, with treatment and time taken as two factors. Other data were evaluated with one-way ANOVA following Dunnett’s multiple comparisons test. *p* < 0.05 was considered significant.

## Results

### DL0410 Could Improve the Memory and Cognition of D-Galactose Induced Aging Mice in Behavioral Tests

#### DL0410 Had No Effect on the Autonomous Activities of Mice

**Figure 2:**
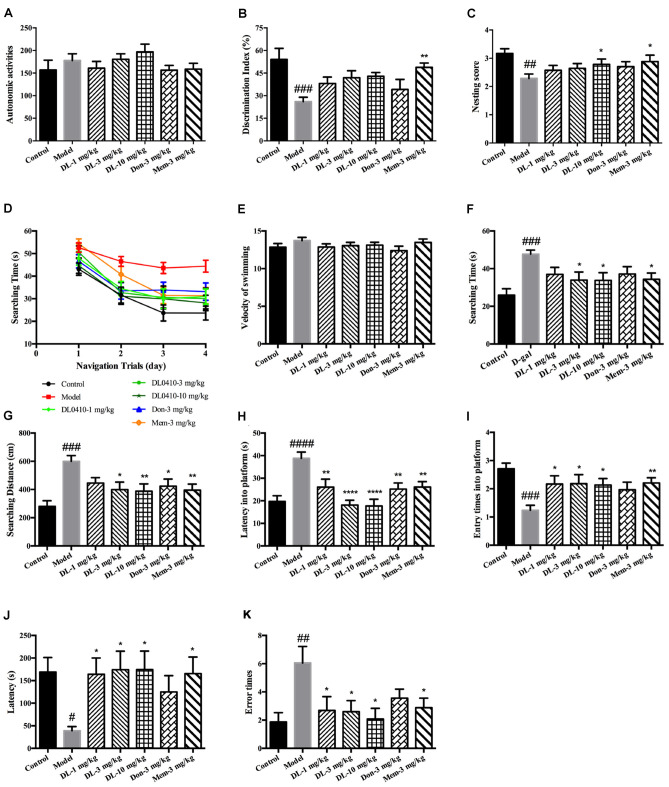
DL0410 could improve memory and cognition of D-galactose-administered mice in behavioral tests. Data are the mean ± SEM (*n* = 16–20). **(A)** There were no differences among groups in the autonomic activities of mice administered D-galactose and DL0410. **(B)** DL0410 tended to increase the discrimination index (DI) of mice in the novel-object recognition test (*F*_(6,83)_ = 3.946, *p* = 0.0016). **(C)** DL0410 could increase the nesting score of mice in the nest-building test (*F*_(6,111)_ = 2.532, *p* = 0.0246). **(D–I)** The effect of DL0410 on the spatial memory of mice in the Morris water maze. **(D)** The searching time of mice decreased as the training time went on in the navigation trials (time effect *F*_(3,446)_ = 39.80, *p* < 0.0001; treatment effect *F*_(6,446)_ = 10.22, *p* < 0.0001). **(E)** DL0410 had no effect on the velocity of swimming of mice. **(F)** DL0410 could shortened the searching time of mice on the fourth day of navigation trials (*F*_(6,113)_ = 3.326, *p* = 0.0047). **(G)** DL0410 could decrease the searching distance of mice on the fourth day of navigation trials (*F*_(6,113)_ = 4.789, *p* = 0.0002). **(H)** DL0410 could decrease the latency of mice crossing the platform in the probe trial (*F*_(6,113)_ = 3.895, *p* = 0.0016). **(I)** DL0410 could increase the entry times of mice crossing the platform in the probe trial (*F*_(6,111)_ = 7.587, *p* < 0.0001). **(J–K)** The effect of DL0410 on the performance of mice in the step-through test. **(J)** DL0410 could prolong the latency of mice stepping into the black chamber (*F*_(6,107)_ = 2.747, *p* = 0.0171). **(K)** DL0410 could reduce the error times of mice stepping into the black chamber (*F*_(6,107)_ = 2.990, *p* = 0.0097). ^#^*p* < 0.05, ^##^*p* < 0.01, ^###^*p* < 0.001, ^####^*p* < 0.0001 vs. control group, *p < 0.05, **p < 0.01, ****p < 0.0001 vs. model group.

Autonomous activities of mice were evaluated before other behavioral tests to exclude the effect of reagents (D-galactose, DL0410, donepezil, memantine) on motor function. There were no obvious differences among the groups (Figure 2A), which indicated that long-term administration of DL0410 and D-galactose did not affect the autonomous activities of mice.

#### DL0410 Tended to Improve the Performance of Mice in the Novel-Object Recognition Test

The novel-object recognition test was undertaken to explore the curiosity of mice about novel objects along with comparison of a familiar object. It also reflected the memory of mice about the familiar object. D-galactose could induce a lower DI compared with the control group (*p* < 0.001), and DL0410 tended to increase the DI of model mice (Figure 2B). Memantine could increase the DI of model mice (*p* < 0.01).

#### DL0410 Could Improve the Nesting Score of Mice in the Nest-Building Test

The nest-building test is used to investigate the activities of daily living of mice. When they are provided with nesting material, they move it to a location in the cage and build up walls. The nest-building result is quantified according to the quality of the nest. Compared with the control group, D-galactose could reduce the nesting score significantly (*p* < 0.01; Figure 2C). Hence, D-galactose could destroy the activities of self-living. DL0410 at 10 mg/kg and memantine could significantly raise the activities of daily life and nesting score at 10 mg/kg (*p* < 0.05 for DL0410 and memantine).

#### DL0410 Could Improve the Spatial Memory of Mice in the Morris Water Maze Test

The Morris water maze is classic test of spatial memory, and involves a navigation trial and probe trial. The results of the navigation trial are shown in Figures 2D–F. On the first day, there was no difference in the searching time in all groups. As training progressed, the searching time of all groups decreased at different rates, and fell to the lowest on the fourth day. We chose the performance on the fourth day as the final results of navigation trials. There were no differences among groups for swimming velocity. D-galactose could prolong the searching time and distance of mice compared with the control group significantly (*p* < 0.001 for both), whereas DL0410 shortened the searching time and distance of model mice remarkably (*p* < 0.05 for searching time at 3 mg/kg; *p* < 0.05 for searching time and *p* < 0.01 for searching distance at 10 mg/kg).

On the fifth day, the platform was removed and the probe trial conducted (Figures 2G–I). The latency for first crossing of the platform of model mice was extended notably (*p* < 0.0001), and the entry times into the platform reduced (*p* < 0.001). DL0410 could shorten the latency into the platform (*p* < 0.01 at 1 mg/kg, *p* < 0.0001 at 3 and 10 mg/kg) and increase the entry times into the platform (*p* < 0.05 at 1, 3 and 10 mg/kg). In this test, the spatial memory of mice was hindered by D-galactose administration, and DL0410 (3 and 10 mg/kg), donepezil and memantine could improve the impaired spatial memory.

#### DL0410 Could Prolong the Latency and Reduce the Error Times of Mice in the Step-Through Test

The step-through test is a type of passive avoidance task in which latency and error times are the two main indices. In the acquisition phase, there were no differences in latency or error times among groups. The results for the retention phase are shown in Figures 2J,K. The latency of the model group was reduced (*p* < 0.05), and the error times increased significantly (*p* < 0.01). In comparison with the model group, DL0410 and memantine could prolong the latency (*p* < 0.05 for all), and reduce the error times (*p* < 0.05 for all).

### DL0410 Tended to Increase the ACh Level and Decrease AChE Activity in the Hippocampus and Cortex

**Figure 3:**
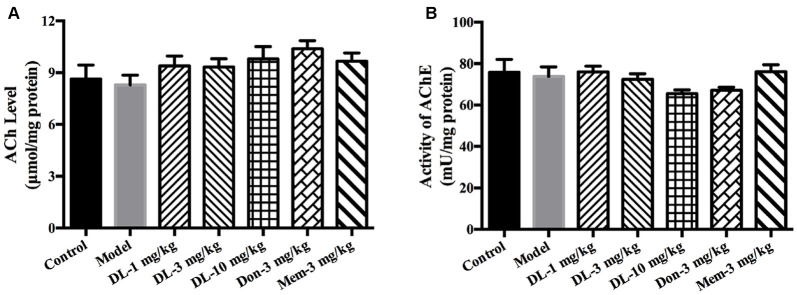
The effect of DL0410 on the cholinergic system in hippocampus and cortex. Data are the mean ± SEM (*n* = 6). **(A)** DL0410 slightly increased the ACh in the hippocampus and cortex. **(B)** DL0410 had a trend to inhibit the activity of Acetylcholinesterase (AChE) in the hippocampus and cortex.

DL0410 is a potent inhibitor of AChE *in vitro*. In the present study, the effect of DL0410 on the cholinergic system in the hippocampus and cortex was investigated. There were no differences between the control group and model group in terms of ACh level or AChE activity (Figure 3). DL0410 (10 mg/kg) and donepezil tended to increase the ACh level and inhibit AChE activity, but memantine had no effect on these two indices.

### DL0410 Could Inhibit Oxidative Damage via Improvement of Antioxidative Defense and Mitochondrial Protection in the Hippocampus and Cortex

In healthy individuals, there is a balance between oxidative and anti-oxidative systems. However, this system is broken if oxidative stress becomes too strong for the anti-oxidative system to overcome, and cellar components (e.g., lipids, proteins) would be subject to damage. The anti-oxidative system consists of reducing substances and anti-oxidative enzymes (catalase, GPx and SOD). ROS, the main oxidant, arises from the damage to the electron-transfer chain in mitochondria. We explored the ameliorating effect of DL0410 on oxidative stress from the aspects of antioxidative defense and mitochondrial protection.

**Figure 4:**
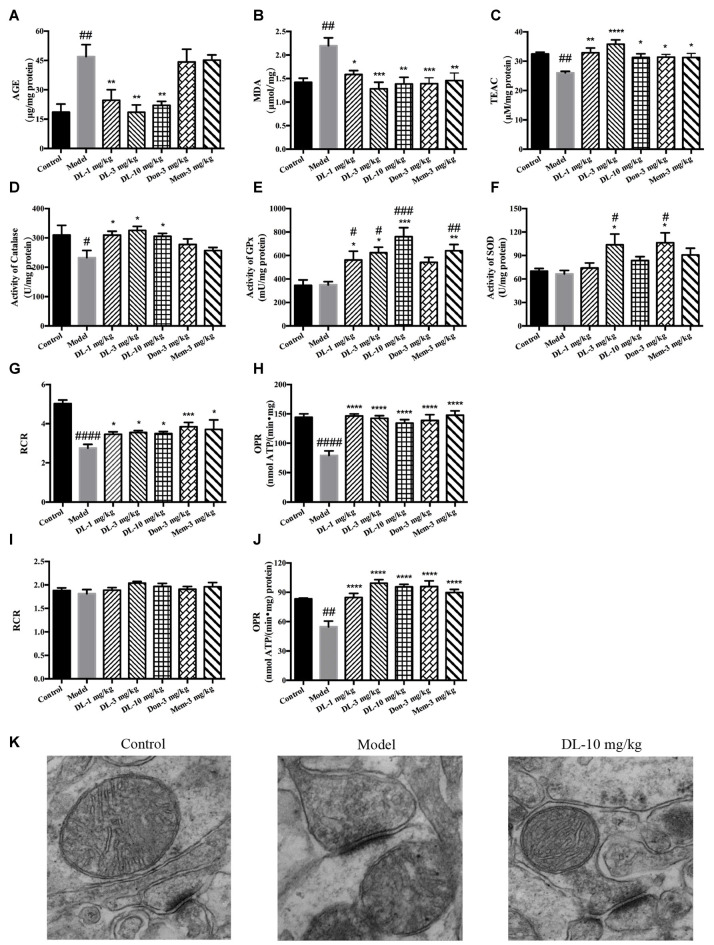
Effect of DL0410 on the oxidative stress, activity of anti-oxidative enzymes and mitochondrial respiration in the hippocampus and cortex. Data are the mean ± SEM (*n* = 5–6). **(A)** DL0410 could reduce advanced glycation end products (AGEs) levels in the hippocampus and cortex (*F*_(6,29)_ = 7.307, *p* < 0.0001). **(B)** DL0410 decreased the production of malondialdehyde (MDA) in the hippocampus and cortex (*F*_(6,29)_ = 5.353, *p* = 0.0008). **(C)** DL0410 could increase the total antioxidant capability of the hippocampus and cortex (*F*_(6,29)_ = 4.900, *p* = 0.0014). **(D)** DL0410 could increase the activity of catalase in the hippocampus and cortex (*F*_(6,27)_ = 2.973, *p* = 0.0232). **(E)** DL0410 could increase the activity of Glutathione peroxidase (GPx) in the hippocampus and cortex (*F*_(6,21)_ = 7.373, *p* = 0.0002). **(F)** DL0410 could increase the activity of superoxide dismutase (SOD) in the hippocampus and cortex (*F*_(6,28)_ = 3.642, *p* = 0.0085). The effect of DL0410 on mitochondrial respiration in the hippocampus and cortex using L-glutamate and L-malate **(G,H)** or succinate **(I,J)** as substrates. Data are the mean ± SEM (*n* = 6–8) **(G,I)** DL0410 could increase the RCR in the hippocampus and cortex (NADH chain *F*_(6,46)_ = 10.46, *p* < 0.0001; FADH_2_ chain *F*_(6,43)_ = 1.397, *p* = 0.2349). **(H,J)** DL0410 could increase the OPR in the hippocampus and cortex (NADH chain *F*_(6,40)_ = 13.21, *p* < 0.0001; FADH_2_ chain *F*_(6,35)_ = 12.49, *p* < 0.0001). **(K)** The representative electron micrograph of mitochondrion in the hippocampus CA1 area (magnification: 150,000×). ^#^*p* < 0.05, ^##^*p* < 0.01, ^###^*p* < 0.001, ^####^*p* < 0.0001 vs. control group, *p < 0.05, **p < 0.01, ***p < 0.001, ****p < 0.0001 vs. model group.

Accumulation of AGEs and MDA (hallmarks of oxidative damage) in the model group was significantly greater than that in the control group (*p* < 0.01 for both; Figures 4A,B). DL0410 could reduce levels of AGEs and MDA (*p* < 0.01 for AGE at 1, 3 and 10 mg/kg, *p* < 0.05 for MDA at 1 mg/kg, *p* < 0.001 for MDA at 3 mg/kg, *p* < 0.01 for MDA at 10 mg/kg), whereas donepezil and memantine reduced only the MDA level (*p* < 0.001 for donepezil and *p* < 0.01 for memantine).

The TAOC of the hippocampus and cortex was investigated further using TEAC as the control (Figure 4C). The TEAC of the model group was lower than that of the control group (*p* < 0.01). DL0410, donepezil and memantine could increase the TEAC level in the hippocampus and cortex (*p* < 0.0001 for DL0410 at 3 mg/kg, *p* < 0.01 for DL0410 at 1 mg/kg, and *p* < 0.05 for DL0410 at 10 mg/kg, donepezil and memantine).

Moreover, we tested the activities of catalase, GPx and SOD in the hippocampus and cortex (Figures 4D–F). The activity of catalase in the model group was significantly lower than that of the control group (*p* < 0.05), whereas the activities of GPx, and SOD in the model group were not affected. In comparison with model mice, DL0410 could improve the activities of catalase, GPx and SOD (*p* < 0.05 for catalase; *p* < 0.05 for GPx at 1 and 3 mg/kg, *p* < 0.001 for GPx at 10 mg/kg; *p* < 0.05 for SOD at 3 mg/kg). Donepezil only improved the SOD notably (*p* < 0.05), and memantine increased the activity of GPx (*p* < 0.01).

The mitochondrial respiration of the hippocampus and cortex was evaluated using L-glutamate and L-malate (NADH chain) or succinate (FADH_2_ chain) as substrates. The improvement of DL0410 on the NADH chain is shown in Figures 4G,H. Long-term administration of D-galactose could decrease the RCR (*p* < 0.0001) and OPR (*p* < 0.0001) in comparison with the control group. DL0410 could improve the RCR (*p* < 0.05 at 1, 3 and 10 mg/kg) and increase OPR (*p* < 0.0001 at 1, 3 and 10 mg/kg). The effect of DL0410 on the FADH_2_ chain is shown in Figures 4I,J. Compared with the control group, D-galactose administration did not affect the RCR, whereas D-galactose could decrease the OPR (*p* < 0.01). DL0410 could increase OPR significantly (*p* < 0.0001 at 1, 3 and 10 mg/kg). Donepezil and memantine could also increase the RCR in the NADH chain and OPR in both chains.

The ultrastructure of mitochondria in the hippocampus was explored further by TEM. Mitochondria in the control group were in an electron-lucent state, with integrated mitochondrial membranes and clearly visible mitochondrial cristae (Figure 4K). In contrast, mitochondria in the model group displayed blurred cristae in electron-dense matrix, and non-integrated mitochondrial membranes. DL0410 (10 mg/kg) could improve the impaired structure of mitochondria remarkably, and the mitochondria showed integrated membranes and clear mitochondrial cristae in electron-lucent matrices.

### DL0410 Ameliorated the Inflammation in the Hippocampus and Cortex via the RAGE/NF-κB Pathway

We have showed that D-galactose could induce AGEs accumulation in the hippocampus and cortex. RAGE (a multi-ligand receptor of AGEs expressed in tissue with high levels of glycol-oxidation) has been reported to be co-localized with activated astrocytes and microglia in the brain. Interaction of AGE with RAGE could activate NF-κB, which would further regulate the expression of pro-inflammatory mediators. In this part of the study, we further investigated the inflammation level in the hippocampus and cortex.

**Figure 5:**
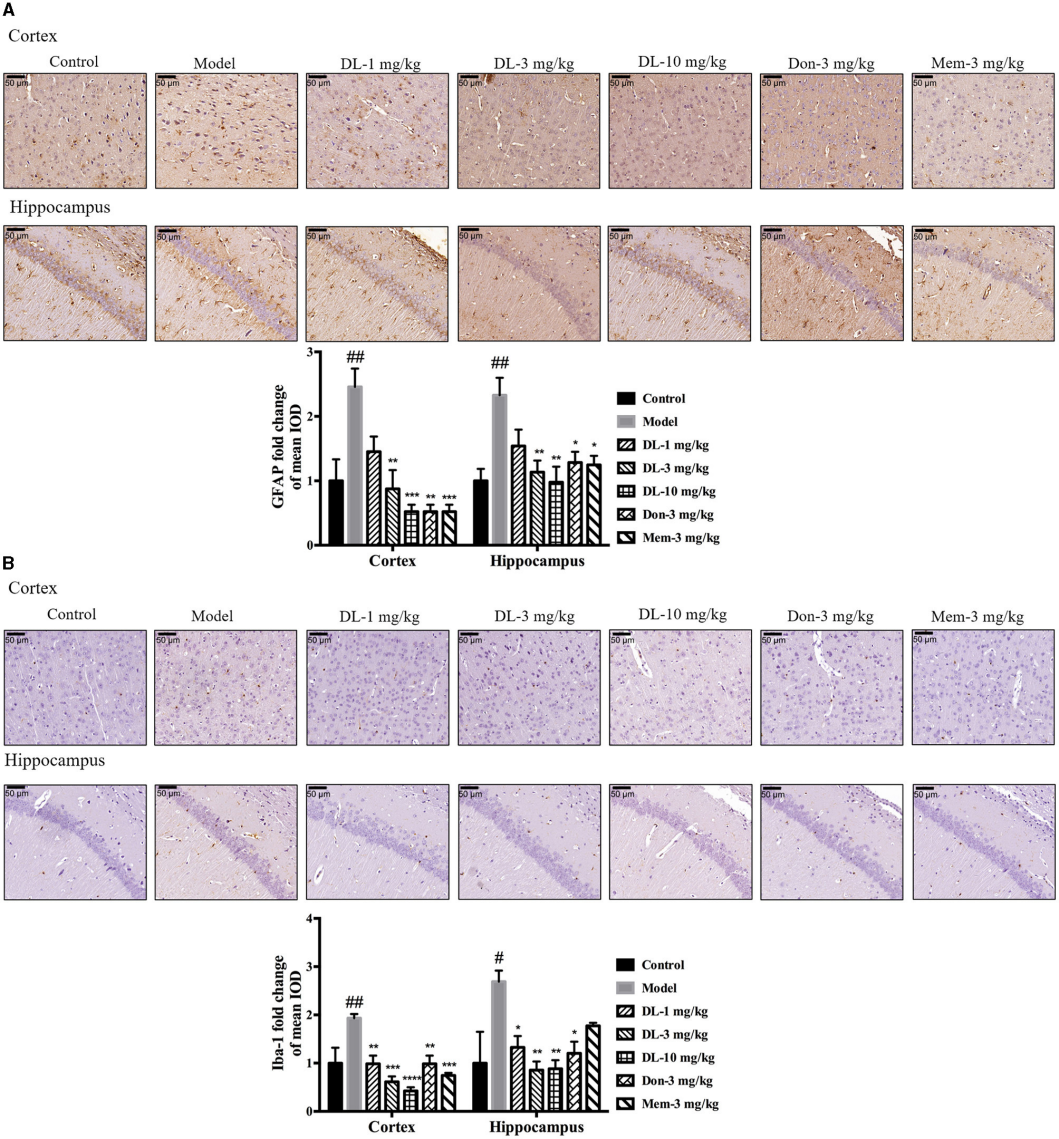
DL0410 decreased the activation of astrocytes and microglia in the hippocampus and cortex. Data are the mean ± SEM (*n* = 3). DL0410 decreased the expression of glial fibrillary acidic protein (GFAP) and Ionized calcium-binding adapter molecule 1 (Iba-1), and decreased the activation of astrocytes **(A)** and microglia **(B)** in the hippocampus and cortex [GFAP: cortex *F*_(6,14)_ = 7.660, *p* = 0.0009, hippocampus *F*_(6,14)_ = 4.969, *p* = 0.0064; Iba-1: cortex *F*_(6,14)_ = 8.685, *p* = 0.0005, hippocampus *F*_(6,13)_ = 4.182, *p* = 0.0146]. Scale bar = 50 μm, and magnification = 400×. ^#^*p* < 0.05, ^##^*p* < 0.01 vs. control group, *p < 0.05, **p < 0.01, ***p < 0.001, ****p < 0.0001 vs. model group.

First, we investigated the status of astrocytes and microglia. The specific markers GFAP and Iba-1 were used to label activated astrocytes and microglia, respectively. In the resting state, astrocytes exhibited a small soma and 3–6 processes, but were activated by D-galactose and resulted in hypertrophy and more processes. The swelling of microglia was found in the activated state. Compared with the control group, the expression of GFAP and Iba-1 in the model group was upregulated significantly (*p* < 0.01 for GFAP in the cortex and hippocampus; *p* < 0.01 for Iba-1 in cortex, *p* < 0.05 for Iba-1 in the hippocampus; Figure 5), denoting activation of astrocytes and microglia in model mice. DL0410 not only inhibited the expression of GFAP in the hippocampus and cortex (*p* < 0.01 for the cortex at 3 mg/kg, *p* < 0.001 for the cortex at 10 mg/kg; *p* < 0.01 for the hippocampus at 3 and 10 mg/kg), but also reduced the expression of Iba-1 in the hippocampus and cortex (*p* < 0.01 for the cortex at 1 mg/kg, *p* < 0.001 for the cortex at 3 mg/kg, *p* < 0.0001 for the cortex at 10 mg/kg; *p* < 0.05 for the hippocampus at 1 mg/kg, *p* < 0.01 for the hippocampus at 3 and 10 mg/kg). Donepezil and memantine inhibited GFAP expression, and donepezil affected Iba-1 expression.

**Figure 6:**
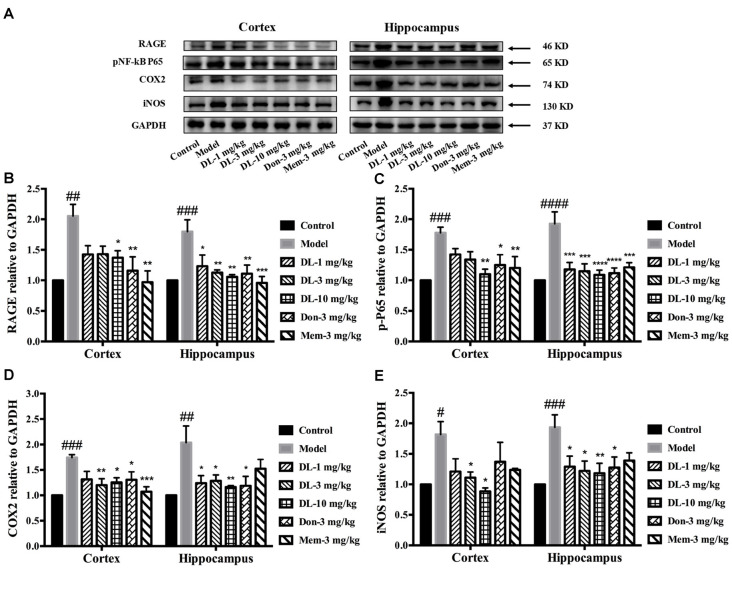
Effect of DL0410 on inflammation via the RAGE/NF-κB pathway in the hippocampus and cortex. Data are the mean ± SEM (*n* = 4–6). **(A)** Representative bands. **(B)** DL0410 could reduce the expression of RAGE in the hippocampus and cortex (cortex *F*_(6,17)_ = 5.012, *p* = 0.0040, hippocampus *F*_(6,21)_ = 5.435, *p* = 0.0016). **(C)** DL0410 could decrease the expression of pNF-κB in the hippocampus and cortex (cortex *F*_(6,28)_ = 5.234, *p* = 0.0010, hippocampus *F*_(6,28)_ = 7.956, *p* < 0.0001). **(D)** DL0410 could decrease the expression of cyclooxygenase-2 (COX2) in the hippocampus and cortex (cortex *F*_(6,27)_ = 5.096, *p* = 0.0013, hippocampus *F*_(6,16)_ = 4.438, *p* = 0.0079). **(E)** DL0410 could decrease the expression of iNOS in the hippocampus and cortex (cortex *F*_(6,13)_ = 2.756, *p* = 0.0592, hippocampus *F*_(6,26)_ = 3.149, *p* = 0.0186). ^#^*p* < 0.05, ^##^*p* < 0.01, ^###^*p* < 0.001, ^####^*p* < 0.0001 vs. control group, *p < 0.05, **p < 0.01, ***p < 0.001, ****p < 0.0001 vs. model group.

Western blotting showed that RAGE expression (Figures 6A,B) in the hippocampus and cortex was upregulated by D-galactose (*p* < 0.01 for the cortex, *p* < 0.001 for the hippocampus). DL0410 reversed the high level of RAGE in the hippocampus and cortex (*p* < 0.05 for the cortex at 10 mg/kg; *p* < 0.05 for the hippocampus at 1 mg/kg, *p* < 0.01 for the cortex at 3 and 10 mg/kg). Western blotting for nuclear transcription factors are shown in Figures 6A,C. p-P65 in the model group was induced to a higher level in the hippocampus and cortex (*p* < 0.001 for the cortex, *p* < 0.0001 for the hippocampus), and DL0410 could notably inhibit the phosphorylation of subunit P65 in the hippocampus and cortex (*p* < 0.01 for the cortex; *p* < 0.001 for the hippocampus at 1 and 3 mg/kg, *p* < 0.0001 for the hippocampus at 10 mg/kg). Donepezil and memantine also decreased the expression of RAGE and p-P65 in the hippocampus and cortex.

Inflammatory mediators were explored further, including COX2 and iNOS (Figures 6A,D,E). D-galactose could induce a higher level of COX2 expression in the hippocampus and cortex (*p* < 0.001 for the cortex; *p* < 0.01 for the hippocampus). DL0410 could decrease COX2 expression significantly (*p* < 0.01 for the cortex at 3 mg/kg, *p* < 0.05 for the cortex at 10 mg/kg; *p* < 0.05 for the hippocampus at 1 and 3 mg/kg, *p* < 0.01 for the hippocampus at 10 mg/kg). Expression of iNOS was also increased (*p* < 0.05 for the cortex, *p* < 0.001 for the hippocampus), and decreased significantly by DL0410 (*p* < 0.05 for the cortex at 3 and 10 mg/kg; *p* < 0.05 for the hippocampus at 1 and 3 mg/kg, *p* < 0.01 for the hippocampus at 10 mg/kg). Donepezil and memantine also inhibited expression of COX2 and iNOS.

### DL0410 Ameliorated the Apoptosis and Synaptic Loss Mediated by ROS-Dependent JNK in the Hippocampus and Cortex

**Figure 7:**
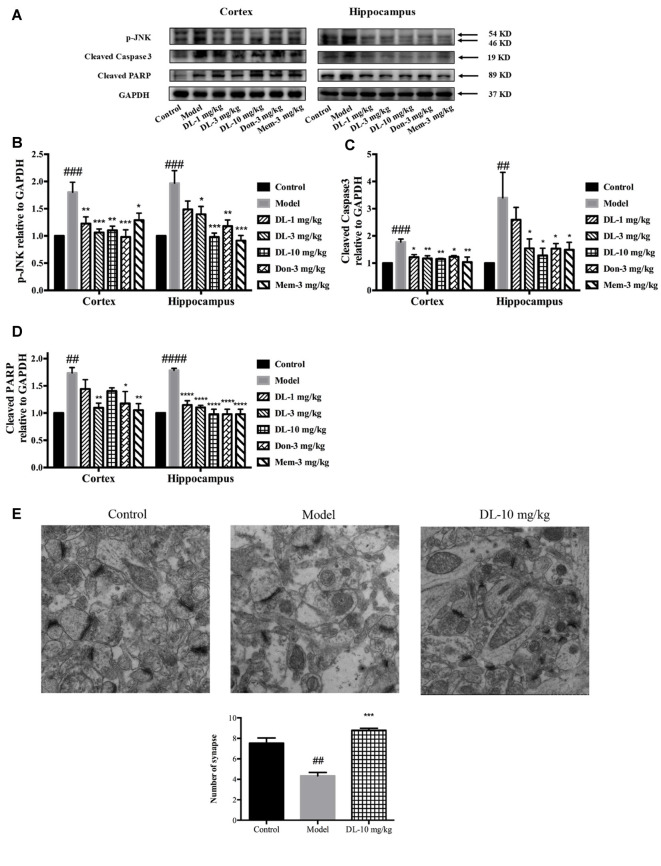
Effect of DL0410 on the expression of p-JNK, cleaved caspase 3 and cleaved PARP in the hippocampus and cortex. Data are the mean ± SEM (*n* = 4–6). **(A)** Representative bands. **(B)** DL0410 could reduce the expression of p-JNK in the hippocampus and cortex (cortex *F*_(6,21)_ = 6.126, *p* = 0.0008, hippocampus *F*_(6,19)_ = 7.427, *p* = 0.0003). **(C)** DL0410 could decrease the expression of cleaved caspase 3 in the hippocampus and cortex (cortex *F*_(6,27)_ = 2.966, *p* = 0.0234, hippocampus *F*_(6,17)_ = 4.158, *p* = 0.0094). **(D)** DL0410 could decrease the expression of cleaved PARP in the hippocampus and cortex (cortex *F*_(6,18)_ = 5.087, *p* = 0.0033, hippocampus *F*_(6,21)_ = 18.89, *p* < 0.0001). **(E)** DL0410 increased the number of synapse in the hippocampus CA1 area (*F*_(2,6)_ = 39.30, *p* = 0.0004). (magnification: 50,000×, *n* = 3). ^##^*p* < 0.01, ^###^*p* < 0.001, ^####^*p* < 0.0001 vs. control group, *p < 0.05, **p < 0.01, ***p < 0.001, ****p < 0.0001 vs. model group.

When oxidative stress and inflammation are increased, JNK known as stress activated protein kinase, would be phosphorylated and involved in apoptosis. Caspase 3, a key executor of apoptosis, is cleaved to an activated form and involved in cell death. We further investigated the level of p-JNK, cleaved caspase 3 and cleaved PARP (Figures 7A–D). Compared with the control group, D-galactose could induce a higher level of p-JNK (*p* < 0.001 for the hippocampus and cortex), cleaved caspase 3 (*p* < 0.001 for the cortex; *p* < 0.01 for the hippocampus) and cleaved PARP (*p* < 0.01 for the cortex; *p* < 0.0001 for the hippocampus) in the hippocampus and cortex. DL0410 was able to inhibit the expression of p-JNK, cleaved caspase 3 and cleaved PARP in the hippocampus and cortex. Donepezil and memantine could inhibit the expression of p-JNK, cleaved caspase3 and cleaved PARP in hippocampus and cortex as well.

Synapses are the basic units for the acquisition and retention of information. Synapses might be subjected to damage when if apoptosis occurs in the brain. TEM was utilized to observe changes in synapses. In comparison with the control group, the number of synapses in the model group was reduced significantly (*p* < 0.01), accompanied by swollen dendritic spines and blurred membranes in presynaptic and postsynaptic ends (Figure 7E). DL0410 could alleviate the swelling of dendritic spines and injured membranes, and increase the number of synapses (*p* < 0.001). The protection afforded by DL0410 to synapses contributes directly to the process of learning.

## Discussion

In this study, we used a D-galactose-induced amnesic model to evaluate the therapeutic effect of DL0410 on the cognitive dysfunction caused by aging. Also, we explored the molecular mechanism of DL0410 from the viewpoint of mitochondrion protection.

Mice had been subjected to 10-week injection of D-galactose (180 mg/kg, s.c.) and 4-week treatment of drug (p.o.) when the behavioral tests were conducted. A series of tests were adopted to evaluate memory and cognitive functions comprehensively. In the autonomous activity test, the motor function of mice in all groups was not affected. Hence, the difference in the other behavioral tests could reflect memory and cognitive functions directly. The Morris water maze is a classic test measuring spatial memory, and mice could find an escape in water via acquired environmental cues (Morris, 1984; Maguire et al., 2003). In the navigation trial, D-galactose could extend the searching time and distance to find the platform. In the probe trial, D-galactose extended the time to cross the platform area (with the platform removed) for the first time, and decreased the entry times into the platform area in 60 s. DL0410 administration could effectively improve the spatial memory, reduce the searching time and distance in the navigation trial, and reduce the latency and increase the entry times of crossing the platform in the probe trial. The step-through test is a type of passive avoidance test, and mice must remember the unpleasant stimulus within the black chamber and refuse to step into it (Pallas et al., 2008). In this test, mice injected with D-galactose behaved badly, stepping into the black chamber more times with shorter latency. However, DL0410 showed a capacity to prevent mice from suffering an electric shock with longer latency and less error times. Nest building is an instinct of mice to protect themselves and also reflects an activity of daily living. This instinct would be lost if they are unwell (Greenberg et al., 2016). In this test, D-galactose-induced aging mice failed to build fine nests, and had a low nesting score. DL0410 could recover the activities of daily living, and make mice build good nests, the scores of which were obviously higher. Novel-object recognition is a non-reward paradigm that can be used to evaluate the non-spatial memory. It involves no punishment, but instead relies on the curiosity of mice about a new object, which is judged by memory (Pitsikas and Tarantilis, 2017). In this test, mice given D-galatose showed a reduced DI, but DL0410 tended to increase the DI (though not significantly). Taken together, the effect of DL0410 on improving impaired memory and cognition was demonstrated by a series of behavioral tests in D-galactose-administered mice. Furthermore, the therapeutic effect of DL0410 could match that of donepezil and memantine at 3 mg/kg, and was even better than them at 10 mg/kg.

The cholinergic system is involved widely in synaptic connections, and forms the basis of information delivery (Schliebs and Arendt, 2006, 2011). Dysfunction of the cholinergic system underlies the defects of memory and cognition (Lim et al., 2015). Amelioration of cholinergic dysfunction is the main mechanism of USA Food and Drug Administration-approved drugs for AD treatment, which have been shown to be effective in alleviating the symptom of dementia clinically (Terry and Buccafusco, 2003; Sanabria-Castro et al., 2017). DL0410 has been shown to be an inhibitor of AChE. Hence, we first evaluated the role of the cholinergic system in the therapeutic effect of DL0410 on D-galactose induced aging mice. D-galactose did not affect the ACh level or AChE activity in the brain. DL0410 (10 mg/kg) tended to inhibit AChE activity and consequently increase the ACh level in model mice only, and its efficacy was similar to that of donepezil. Therefore, the D-galactose-administered model might not imitate cholinergic dysfunction, and AChE inhibitors have been demonstrated to affect normal cholinergic transmission slightly. Hence, another mechanism of action of DL0410 must be operating in the D-galactose induced aging mice.

The neurotoxicity of D-galactose arises mainly from oxidative damage. Levels of MDA (a hallmark of oxidative damage in lipids) and AGEs (direct markers of damage by D-galactose or oxygen free radicals to proteins) were significantly higher in the brains of D-galactose administered mice, which confirmed the higher level of oxidative stress. Levels of the free radical-scavenger catalase were decreased by D-galactose, but the activity of GPx and SOD were unchanged compared with the control group. DL0410 reduced the oxidative damage in cellular components (MDA and AGEs) and consolidated anti-oxidative defenses by increasing the TAOC and activities of catalase, GPx and SOD.

The brain is an organ of high energy demand, and needs 25% glucose for energy metabolism (Rossi et al., 2001). Mitochondrion, as the energy plant in the cell, consumes about 90% of the oxygen of a cell, which is used for ATP production. Hence, mitochondria are also the major sources of oxygen free radicals in cells (Figueira et al., 2013; Li et al., 2016). However, mitochondria are also the targets of oxygen free radicals, leading to a vicious circle between mitochondrial damage and free-radical production (Green and Kroemer, 2004; Vakifahmetoglu-Norberg et al., 2017). A series of evidences have reported that the activities of complexes involved in the electron-transfer chain and mitochondrial structure are damaged by long-term administration of D-galactose (Parker et al., 1994; Kumar et al., 2011; Prakash and Kumar, 2013; Du et al., 2015). Hence, we next detected the function of mitochondria and oxygen consumption in mitochondrial respiration. The RCR is the ratio of V3 to V4, the velocity of oxygen-atom consumption in the presence of ADP and after the exhaustion of ADP, respectively. The OPR is representative of the velocity of ATP production. In the NADH chain, D-galactose could decrease the RCR and OPR, whereas D-galactose could reduce the OPR in the FADH_2_ chain. That is, oxidative phosphorylation and ATP production in mitochondria were damaged by D-galactose. However, DL0410 could recover this impaired mitochondrial function, and increase the RCR and OPR. Hence, there was no obstacle in the process of oxidation, phosphorylation and their coupling, which reflected the high efficiency of ATP production, and could be attributed to the complexes and substrates in the electron-transfer chain. Furthermore, TEM was used to investigate the ultrastructure of mitochondria. We found that mitochondria in the model group displayed obvious structural damage, with electron-dense matrices as well as injured membranes and cristae. However, DL0410 (10 mg/kg) could protect mitochondrial structure from damage, which might be the basis of normal mitochondrial function. Therefore, DL0410 inhibited oxidative stress via an increase in antioxidative defense, on the other hand, DL0410 was found to be protective for mitochondria, cutting off the vicious circle of free radical production. The latter might be the primary effect of DL0410.

Neuroinflammation starts before the occurrence of cognitive impairments, and this process would co-evolve with deterioration of cognitive defects and dementia from preclinical to clinical stages (Raj et al., 2017). Thus, neuroinflammation has been regarded as a vital aspect in relieving dementia (Cuello, 2017). RAGE is a multi-ligand receptor with high affinity for AGE-modified proteins (e.g., Aβ), and locates in neurons, astrocytes and microglia in the brain (Wendt et al., 2003). RAGE can be activated by AGEs and trigger strong oxidative damage and inflammation (Singh et al., 2001). NF-κB is a free radical-sensitive nuclear transcription factor for inflammation pathway, could be activated directly by free radicals, or indirectly by AGEs binding to RAGE. In the present study, astrocytosis and microgliosis were induced significantly by D-galactose, as indicated by high expression of GFAP and Iba-1 in the cortex and hippocampus. Furthermore, D-galactose could induce significantly higher expression of RAGE and p-NF-κB P65, with the latter translocating to the nucleus to regulate the expression of pro-inflammatory mediators. COX2 and iNOS, participating in the synthesis of prostaglandin-E2 and nitric oxide, were found to be highly expressed. However, DL0410 administration could significantly inhibit the activation of astrocytes and microglia and the RAGE/NF-κB P65 signaling pathway, leading to a reduction in expression of COX2 and iNOS. Therefore, DL0410 could suppress the inflammation induced by D-galactose in the cortex and hippocampus.

JNK, a stress-activated protein kinase, can be activated under the condition of oxidative stress and inflammation (Tournier et al., 2000; McManus and Franklin, 2016). JNK can also initiate the apoptosis pathway via the release of pro-apoptotic factors (Dai et al., 2017). Pro-apoptotic factors further help to form the apoptosome (cytochrome c, Apaf-1, procaspase 9 and dATP), which initiates the caspase cascade and destroys PARP through caspase 3. We found that p-JNK expression was induced to be higher in D-galactose administered mice, and that caspase 3 was activated with PARP obviously cleaved. Hence, D-galactose actually induced a higher level of apoptosis in the brain. DL0410 treatment could inhibit apoptosis by suppressing the phosphorylation of JNK and activation of caspase 3, and subsequently reduced the level of cleaved PARP. Since synapses are the main units for cognition, it might suffer from loss when apoptosis occurs in the brain. Further loss of synapses in the CA1 area of the hippocampus has been notable in the model group via TEM, and DL0410 could increase the number of synapses and protect dendrite structure. Thus, the inhibition of apoptosis and synaptic protection afforded by DL0410 could be the direct factor for the improvement of memory and cognition.

Mitochondrial dysfunction is a hallmark and a driver of aging (López-Otín et al., 2013). When mitochondria are damaged, the efficacy of the respiratory chain diminishes, with increasing loss of electrons and decreasing ATP production. An enhanced level of ROS aggravates cellular damage and facilitates release of pro-inflammatory mediators, which in turn accelerate aging (Korolchuk et al., 2017). Oxidative stress, inflammation and apoptosis are three pathogenic factors in the process of aging, and also participate in the synaptic loss and dementia induced by D-galactose.

**Figure 8:**
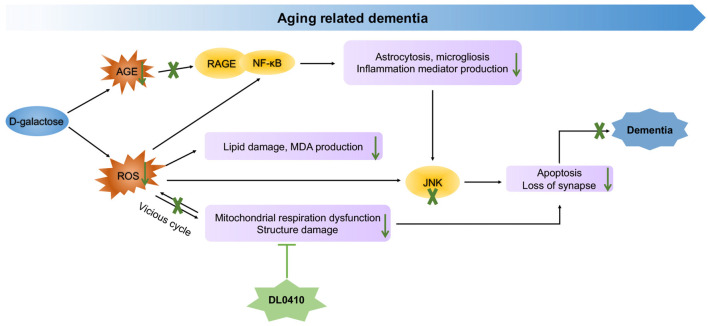
Molecular mechanism of DL0410 in the improvement of dementia induced by D-galactose.

In the present study, the primary protection for mitochondrial by DL0410, inhibited ROS accumulation and neuroinflammation induced by ROS and AGEs, and the final protection for neurons and synapses contributed to cognitive improvement (Figure 8). However, inhibition of AChE activity was not notable in this model. Therefore, mitochondrial protection appears to be the main and key target of DL0410 in this model, which has never been reported before, and inhibition of neuroinflammation and apoptosis are also two important parts. With regard to the treatment of complicated disease, such as AD and PD, multiple targeted agents are a novel trend (Korábecný et al., 2017). DL0410, an AChE inhibitor, that offers mitochondrial protection as well as anti-oxidant, anti-inflammatory, anti-apoptosis and synaptic-protective properties, might be a promising agent for AD therapy.

In sum, DL0410 could ameliorate the cognitive impairments in a series of behavioral tests in D-galactose induced aging mice. This therapeutic effect of DL0410 could be attributed to protection of mitochondria in the cortex and hippocampus, which subsequently attenuated oxidative damage and inflammation, and reduced neuronal and synaptic loss.

## Author Contributions

WL, AL and GD designed this study and wrote the manuscript; HJ, LX, WZ and DK carried out the experiments; WL and WZ analyzed data. All authors listed, have made substantial, direct and intellectual contribution to the work, and approved it for publication.

## Conflict of Interest Statement

The authors declare that the research was conducted in the absence of any commercial or financial relationships that could be construed as a potential conflict of interest.
