# Corrigendum: Multi-protection of DL0410 in ameliorating cognitive defects in D-galactose induced aging mice

**DOI:** 10.3389/fnagi.2024.1419861

**Published:** 2024-06-07

**Authors:** Wenwen Lian, Hao Jia, Lvjie Xu, Wei Zhou, De Kang, Ailin Liu, Guanhua Du

**Affiliations:** State Key Laboratory of Bioactive Substance and Function of Natural Medicines, Institute of Materia Medica, Chinese Academy of Medical Sciences and Peking Union Medical College, Beijing, China

**Keywords:** DL0410, Alzheimer's disease, mitochondrion, oxidative stress, neuroinflammation, apoptosis, synaptic protection

In the published article, there was an error in Figure 5 as published. The representative pictures for GFAP staining in the hippocampus were identical for both model group and Don-3 mg/kg group, in which the picture in Model group was used by mistake. The corrected Figure 5 and its caption appear below.

**Figure 5:**
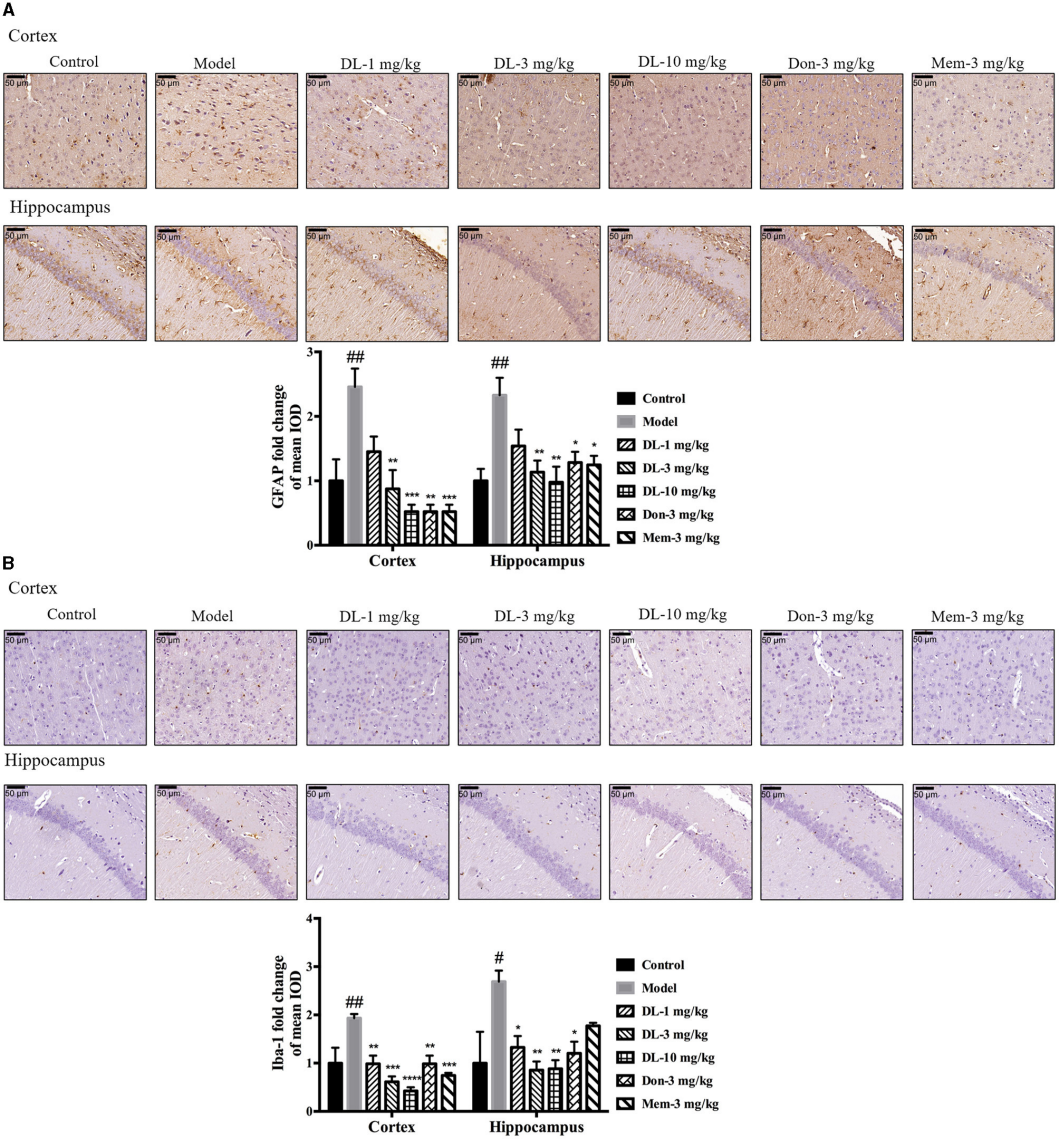
DL0410 decreased the activation of astrocytes and microglia in the hippocampus and cortex. Data are the mean ± SEM (*n* = 3). DL0410 decreased the expression of glial fibrillary acidic protein (GFAP) and Ionized calcium-binding adapter molecule 1 (Iba-1), and decreased the activation of astrocytes **(A)** and microglia **(B)** in the hippocampus and cortex [GFAP: cortex *F*_(6, 14)_ = 7.660, *p* = 0.0009, hippocampus *F*_(6, 14)_ = 4.969, *p* = 0.0064; Iba-1: cortex *F*_(6, 14)_ = 8.685, *p* = 0.0005, hippocampus *F*_(6, 13)_ = 4.182, *p* = 0.0146]. Scale bar = 50 μm, and magnification = 400 × . ^#^*p* < 0.05, ^##^*p* < 0.01 vs. control group, ^*^p < 0.05, ^**^p < 0.01, ^***^p < 0.001, ^****^p < 0.0001 vs. model group.

The authors apologize for this error and state that this does not change the scientific conclusions of the article in any way. The original article has been updated.

